# Caretakers’ stated willingness to pay for children’s spectacles in cross river state, Nigeria and its implication for a cross-subsidisation scheme: a cross-sectional study

**DOI:** 10.1186/s12889-023-15901-7

**Published:** 2023-06-05

**Authors:** Anne Effiom Ebri, Ciaran O’Neill, Kenneth Azubuike, Nathan Congdon, Christine Graham, Lynne Lohfeld, Ving Fai Chan

**Affiliations:** 1 Charis Vision & Health Mission, Calabar, Nigeria; 2 Centre for Public Health, School of Medicine, Dentistry and Biological Sciences, Institute of Clinical Science, Queen’s University Belfast, Block A, Royal Victoria Hospital, BT12 6BA Northern Ireland, UK; 3 Zhongshan Ophthalmic Centre, Sun Yat-sen University, Guangzhou, China; 4 College of Health Sciences, University KwaZulu Natal, Durban, South Africa

**Keywords:** Willingness to pay, Spectacles, Children, Refractive error, Cross-subsidisation

## Abstract

**Background:**

Understanding caretakers’ willingness to pay (WTP) for their children’s spectacles is essential to improving the sustainability of refractive error services and spectacle provision. Therefore, we investigated the willingness of caretakers to pay for their children’s spectacles in a multi-centre study to develop a spectacle cross-subsidisation scheme in the Cross River State (CRS), Nigeria.

**Methods:**

We administered the questionnaire to all caretakers whose children were referred from school vision screenings to four eye centres for full refraction assessment and dispensing of corrective spectacles from 9 August to 31 October 2019. We collected information on socio-demography, children’s refractive error types, and spectacle prescription and then asked the caretakers about their WTP for the spectacles using a structured questionnaire and bidding format (in the local currency, Naira, ₦).

**Results:**

A total of 137 respondents (response rate = 100%) from four centres were interviewed: with greater proportion of women (n = 92, 67.1%), aged between 41 and 50 years (n = 59, 43.1%), government employees (n = 64, 46.7%) and had acquired college or university education (n = 77, 56.2%). Of the 137 spectacles dispensed to their children, 74 (54.0%) had myopia or myopic astigmatism (equal to or greater than 0.50D). The mean stated WTP for the sample population was ₦3,560 (US$ 8.9) (SD ± ₦1,913.4). Men (p = 0.039), those with higher education (p < 0.001), higher monthly incomes (p = 0.042), and government employees (p = 0.001) were more willing to pay ₦3,600 (US$9.0) or more.

**Conclusion:**

Combining our previous findings from marketing analysis, these findings provided a basis to plan for a children’s spectacles cross-subsidisation scheme in CRS. Further research will be needed to determine the acceptability of the scheme and the actual WTP.

## Background

With an estimated population of 173 million [1], children between 0 and 15 years old constitute about 45% of the population, of whom 52 million are in primary and secondary schools in Nigeria. [2] School-age children’s most common health conditions, such as malnutrition and mental health issues, negatively affect their education, impact cognition and learning, reduce school enrolment and increase absenteeism. [3] In addition, uncorrected refractive error is one of the main causes of vision impairment in school children [4] and negatively impacts children’s learning and education. In Nigeria, the refractive error prevalence among children ranged from 1.9% in South-East^5^ to 8.0% in North-Central region. [6] The prevalence is increasing [5–10], with most children who need spectacles not owning them. [10].

Nigeria is one of the largest economies in Africa [11] making the cost recovery mechanism a potential solution to address the issue of unaffordable refractive services among the poor. For example, the L V Prasad Eye Institute [12] and Aravind Eye Hospital [13] have successfully used cross-subsidisation strategies to deliver equitable eye health services to their populations. While it has been used in delivering presbyopic glasses in Timor Leste, [14] the cost recovery mechanism for dispensing affordable spectacles for children has not received much attention. Non-Governmental Organisations have supported dispensing free spectacles through a funded programme in Nigeria since 2006. However, the need to consider and initiate cost recovery measures is hampered by the paucity of data for government and non-government agencies to set up systems to supply affordable and equitable spectacle services.

Cross River State (CRS) is one of the 36 States in Nigeria, with about 3 million population [15], and among the least resourced states in the country. [16] The state is divided into three health administrative zones. Each zone has at least one eye clinic recently renovated to accommodate a dedicated child eye unit, funded by the *Seeing is Believing* project in collaboration with the CRS Ministry of Health. The project provided free custom-made spectacles to all children who needed them from 2017 to 2019. However, future programmes relying on the free distribution of spectacles are not sustainable. There has not been structured pricing for spectacles, and the centres remain heavily reliant on market vendors for their supply. Hence, a pricing structure can facilitate a sustainable spectacle provision scheme incorporated in primary eye care service delivery.

In 2019, the CRS Ministry of Health considered a spectacle cross-subsidisation scheme, where children from poorer communities can procure lower-cost spectacles whose price is subsidised through the profits made from selling mid-and high price range spectacles. This scheme has been described in detail in a previous publication. [17] The scheme aims to (i) increase access to spectacles among the urban poor and rural poor, (ii) relieve the Ministry from the unsustainable donor-reliant free spectacles programme, and (iii) fully utilise the dispensing units to achieve economies of scale. Hence, a series of market research studies were conducted in 2019 to understand the demography profile and ocular needs of the children attending the local child eye clinics [17], factors affecting caretakers’ when purchasing their children’s spectacles [18], the reasons that prevented the caretakers from bringing their children from attending a follow-up eye exam [19] and the caretakers’ willingness to pay for their children’s spectacles (which is a the focus of this article). We found that nearly 50% of the 3799 children who visited an eye clinic in CRS needed and procured spectacles, and almost all of them (90%) were first-time buyer; [17] 60% were urban- and rural-poor children; [17] unaffordability of spectacles was the main reason for not pursuing follow-up examination after they failed vision screening [19]; and good frames and lenses were priority features for parents who could afford to buy spectacles for their children. [18].

To ensure a capital-efficient cross-subsidisation scheme, pricing also needs to be guided by knowledge of the consumers’ willingness to pay (WTP) for quality refractive error services and the factors influencing their decisions. It is particularly useful to understand the consumers’ values related to the service and predict their purchase behaviour to ascertain the financial viability of commercial provision and the price at which to “pitch” a service. [20] Perceived need, values, knowledge, treatment experience, and consumers’ socio-demographic profiles are shown to influence WTP for a pair of spectacles in Cambodia [21] and Ethiopia. [22] Hence, the current study aims to assess the willingness of caretakers to pay for their children’s spectacles in four centres in Cross River State to plan an evidence-based spectacle cross-subsidisation scheme in Nigeria as part of improved eye care programmes for children.

## Methods

This is a cross-sectional study conducted among four child eye clinics in CRS. This study formed part of the 2019 market research study. Caretakers (parents or guardians) who brought their children to the child eye health clinics in the metropolis of Calabar and semi-urban areas of Ugep, Ikom and Ogoja for full refractive error services from 9 August to 31 October 2019 were invited to participate in an interview-based survey. Trained local observers administered the interview using a pre-tested questionnaire. The lowest and the highest market prices were determined through a market survey. Five starting prices and incremental/decremental intervals of ₦ 250 (US$0.63) were established through discussions with key personnel at the national Ministry of Health to understand the local pricing method familiar to the study population.

The WTP survey was conducted in a private room by an interviewer after obtaining informed consent from the respondents. To ensure that the respondents could appropriately contextualise the valuation of the pair of spectacles for their children and to reduce the potential for strategic bias, the following information was provided: (a) their child’s reduced vision was caused by significant refractive error, and according to the optometrist, spectacles would effectively manage the cause, (b) the child’s vision would improve with the spectacle wear and (c) responses to questions regarding WTP for spectacles would not affect in any way the quality of the spectacles their child receives.

The study adopted the bidding format, which is an effective method of bargain used locally to determine the price of a pair of spectacles. [13] The binary bidding format utilised was explained. The respondents were first asked if they would pay for their children’s spectacles. If the respondent was unwilling to pay for the pair of spectacles for the child, the WTP was recorded as zero, and the reason for not being willing to pay for the child’s spectacles was reported. If the respondent could not give a specific amount they would pay, the interviewer randomly drew a card that listed a starting price for the respondent. The random starting point was intended to reduce starting point bias. The respondent was then asked whether they would pay the amount shown. If the respondent’s response was yes, the interviewer then increased the amount by ₦250 (US$ 0.6) in an iterative fashion. Increasing the price by the same amount continued until the respondent indicated they were unwilling to pay the stated amount. If the respondent’s initial response to the starting price was no, the interviewer decreased the amount by ₦250 (US$0.6), and the process continued iteratively in decrements of ₦250 (US$0.6) until the respondent gave a positive answer. The maximum price the respondent was willing to pay was then recorded.

The study data were transferred to Microsoft Excel and uploaded onto Statistical Package for Social Sciences 26–2018 for analysis. Descriptive analysis was carried out for the demographic variables, and the maximum WTP data were analysed. WTP was the maximum amount a caretaker was willing to pay for the child’s spectacles. Univariate analyses were performed using chi-square tests to provide crude associations of socio-demographic factors and WTP of ₦3,600 (US$9.0). In cases where more than 20% of the cells have expected frequencies less than 5 (such as comparing centre and occupation of caretakers who were willing to pay ₦3600 or more for child’s spectacles in the future), Fisher’s Exact tests were used instead. The absolute value of the refractive error was defined as hyperopia (+ 0.75 Dioptre [D] or worse), with or without astigmatism, myopia (− 0.50D or worse), with or without astigmatism and emmetropia (≤ 0.50D to < − 0.50D) with or without astigmatism. The Naira to United States (US) Dollar exchange rate used in the study was ₦400.00 to 1 US Dollar (Xe Currency Converter as of October 2019).

## Results

### Respondents

Of the 137 respondents, 53 (38.7%) were from Ugep, 21 (15.3%) from Ogoja, 35 (25.6%) from Ikom and 28 (20.4%) from Calabar (response rate = 100%). The larger proportion of the respondents was female (n = 92, 67.1%), aged between 41 and 50 years (n = 59, 43.1%) and have never owned a pair of spectacles (n = 71, 52.2%). Over 40% (n = 55) of the households earned a monthly income of more than ₦50,000 (US$125). Government employment (n = 64; 46.7%) was the most common occupation of the principal household income earners, followed by private/self-employment (n = 51, 37.2%). Over half of the respondents (n = 77; 56.2%) had completed college or university education. The majority of the children involved were under 12 years of age (n = 79; 58.1%), female (n = 77; 56.2%) and had myopia with or without astigmatism (n = 74; 54.0%) (see Table 1).

**Table 1:** Demography of Study Respondents

Characteristics	Number of respondents (%)	95% confidence interval
**Clinics**		
Ugep Ogoja Ikom Calabar	53 (38.7%) 21 (15.3%) 35 (25.6%) 28 (20.4%)	38.8 − 47.2 9.0–21.0 18.6–33.3 13.3–26.7
**Caretakers’ Gender**		
Male Female	45 (32.9%) 92 (67.1%)	25.1–40.8 59.2–74.9
**Caretakers’ age (years)**		
≤ 40 41–50 ≥ 51	55 (40.2%) 59 (43.1%) 23 (16.7%)	31.8–48.2 34.7–51.3 10.5–22.9
**Caretakers’ gross household income**		
≤ ₦50,000 > ₦50,000 Don't know	67 (48.8%) 55 (40.2%) 15 (11.0%)	40.4–57.2 32.0–48.4 5.76–16.2
**Caretakers’ highest education attained**		
Primary school completed Secondary school completed University/College completed No schooling	23 (16.8%) 30 (21.9%) 77 (56.2%) 7 (5.1%%)	10.5–23.1 15.0–28.8 47. 9–64.5 1.4–8.8
**Caretakers’ occupation**		
Government employed Agriculture/factory Retired/Unemployed/Housewife Private company/Self-employed/Others	64 (46.7%) 19 (13.8%) 3 (2.3%) 51 (37.2%)	38.3–55.1 8.0–19.6 0.0–4.8 29.1–45. 3
Caretakers’ spectacle-wear history*		
Yes No	65 (47.5%) 71 (51.8%)	39.4–56.2 43.8–60.6
**Child’s Refractive error**		
Myopia with and without astigmatism Hyperopia with and without astigmatism Astigmatism only	74 (54.0%) 26 (19.0%) 37 (27.0%)	45.6–62.3 12.4–25.6 19.6–36.4
**Child’s gender**		
Male Female	60 (43.8%) 77 (56.2%)	35.5–55.1 47.9–64.5
**Child’s Age**		
< 12 years ≥ 12 years	79 (58.1%) 57 (41.5%)	49.8–66.4 33.2–49.8

*Missing data = 1

**Stated willingness to pay (WTP)** ₦**3,600 for a pair of spectacles**.

The mean WTP for the sample population was ₦3,560 (SD ± 1913.4) (US$ 8.9), approximated to ₦3,600 (US$9.0). The proportion of caretakers in the study population willing to pay ₦3,600 (US$9.0) or more for their children’s spectacles in the future was 47.4% (95% CI 39% − 55.8%).

The mean WTP for the sample population was the lowest in Ikom (₦3142.9 or US$7.86, 95%CI 2488.2–3797.6) and from caretakers engaging in agricultural/factory activities (₦3215.4 or US$8.04, 95%CI 2643.1–4381.6) while highest from caretakers who earned more than ₦50,000 or US$125 per month (₦4195.5 or US$10.5, 95%CI 3695.3–4695.7). Caretakers were also willing to pay highest if their children had myopia with and without astigmatism (₦3918.2 or US$9.8, 95%CI 3437.1–4399.5), followed by astigmatism only (₦2917.6 or US$7.29, 95%CI 2428.2–3406.9) and hyperopia with and without astigmatism (₦2807.7 or US$7.02, 95%CI 2815.3–3430.4). (Table 2) Four caretakers reported unwillingness to pay any amount for their children’s spectacles. The reasons were that donor agencies would always provide their spectacles and that young children would outgrow them.

**Table 2:** Mean willingness-to-pay among respondents

Characteristics	Mean WTP (Naira)	95% confidence interval
**Centre**		
Ugep Ogoja Ikom Calabar	3708.5 3535.7 3142.9 3821.4	3185.9–4231.1 2823.0–4248.4 2488.2–3797.6 3123.1–4519.8
**Caretakers’ gender**		
Male Female	3557.1 3560.6	2994.4–4119.9 3170.0–3951.1
**Caretakers’ age (years)**		
≤ 40 41–50 ≥ 51	3542.7 3557.4 3504.5	3038.7–4046.6 3067.9–4046.8 2693.3–4315.7
**Caretakers’ gross household income**		
≤ ₦50,000 > ₦50,000 Don't know	3558.3 4195.5 3486.8	3094.9–4021.6 3695.3–4695.7 2416.0 -4522.7
**Caretakers’ highest education attained**		
Primary completed Secondary completed University/College completed No schooling	3415.1 3527.2 3560.6 3721.6	2627.6–4202.6 2840.8 -4213.3 3133.7–3987.5 2202.6–5240.5
**Caretakers’ occupation**		
Government employed Agriculture/factory Retired/Unemployed/Housewife Private company/Self-employed/Others	3546.8 3215.4 3470.6 3580.7	3077.9–4015.4 2643.1–4381.6 1370.4–5570.8 3057.8–4103. 7
**Caretakers’ spectacle wear history**		
Yes No	3557.4 3542.7	3091.1–4023.6 3099.2–3986.2
**Child’s Refractive error**		
Myopia with and without astigmatism Hyperopia with and without astigmatism Astigmatism only	3918.2 2807.7 2917.6	3437.1–4399.5 2815.3–3430.4 2428.2–3406.9
**Child’s gender**		
Male Female	3566.9 3560.6	3084.2–4049.6 3135.7–3987.5
**Child’s Age**		
< 12 years ≥ 12 years	3560.6 3539.3	3139.1–3982.1 3042.5–4036.0

Male caretakers (p = 0.039), with a college education (p < 0.001), a government employee (p < 0.001, and monthly income more than ₦50,000 (p = 0.042) were significantly willing to pay ₦3,600 (US$9.0) or more. Majority female caretakers (58.4%), with no schooling (85.7%), working in the agriculture or factory workers (78.9%) were unwilling to pay more than ₦3,600 (US$9.0). (Table 3)

**Table 3:** **Willngness to pay** ₦**3600 or more for child’s spectacles in the future**

Characteristics	WTP ≥ ₦3600		P value
	Yes	No	
**Centre**			
Ugep Ogoja Ikom Calabar	32 (60.4) 8 (38.1) 12 (34.3) 13 (46.4)	21 (39.6) 13 (61.9) 23 (65.7) 15 (53.6)	0.081
**Caretakers’ gender**			
Male Female	27 (60.0) 38 (41.3)	18 (40.0) 54 (58.4)	0.039
**Caretakers’ age (years)**			
≤ 40 41–50 ≥ 51	29 (52.7) 26 (44.1) 10 (43.5)	26 (47.3) 33 (55.9) 13 (56.5)	0.597
**Caretakers’ gross household income**			
≤ ₦50,000 > ₦50,000 Don't know	31 (46.3) 31 (60.0) 3 (20.0)	36 (52.2) 24 (40.0) 12 (80.0)	0.042
**Caretakers’ highest education attained**			< 0.001
Primary completed Secondary completed University/College completed No schooling	8 (34.8) 13 (43.3) 43 (55.8) 1 (14.3)	15 (65.2) 17 (56.7) 34 (44.2) 6 (85.7)	
**Occupation**			< 0.001
Government employed Agriculture/factory Retired/Unemployed/Housewife Private company/Self-employed/Others	35 (54.7) 4 (21.1) 2 (66.7) 24 (47.1)	29 (45.3) 15 (78.9) 1 (33.3) 27 (52.9)	
Caretakers’ spectacle wear history*			0.154
Yes No	35 (53.8) 30 (41.7)	30 (46.2) 42 (58.3)	
**Child’s refractive error type**			
Myopia with or without astigmatism Hyperopia with or without astigmatism Astigmatism only	41(55.4) 10 (38.5) 14 (37.8)	33(44.6) 16 (61.5) 23 (66.2)	0.129
**Child’s gender**			
Male Female	33 (55.0) 32 (41.6)	27 (45.0) 45 (58.4)	0.118
Child’s age* (years)			
< 12 ≥ 12	40 (50.6) 24 (42.1)	39 (49.4) 33 (57.9)	0.326

*Missing data = 1

## Discussions

Our study determined caretakers’ willingness to pay (WTP) for their children’s spectacles in four clinics in CRS, Nigeria. The findings showed that caretakers were willing to pay a mean stated WTP of ₦3,600 (US$ 9.0), where men, caretakers with higher education, higher monthly incomes and government employees were more willing to pay ₦3,600 (US$ 9.0) or more. A majority of them had never owned a pair of spectacles. Together with our previous work, these findings can support the development of a spectacle cross-subsidisation scheme in CRS by the Ministry of Health.

The caretakers were willing to pay a mean of ₦3,600 (US$ 9.0) for their children’s spectacles, an amount higher than ₦3,000 (US$7.50), which is the mean cost for a pair of custom-made spectacles for children locally. With nearly half of the caretakers willing to pay ₦ 3,600 (US$ 9.0) or more, the remaining half could benefit from lower-priced spectacles. Furthermore, based on our previous market research, we identified that the children who attended the four child eye health clinics were as follows: 40% affluent, 32% rural poor and 28% urban poor. [17] However, local stakeholders indicated that since the cessation of the free spectacle delivery programme, the proportion of rural and urban poor children purchasing spectacles from their clinic had reduced to 20%. Faderin et al. [9] also reported the main constraint for spectacle uptake was non-affordability and economic barriers. Taking these findings into account, a cross-subsidisation scheme can increase the reach, where over half of the caretakers unwilling to pay the stated amount can be offered lower-priced spectacles for their children.

Our study also showed four caretakers who were unwilling to pay any amount. They were from semi- rural and rural area whose caretakers had lowest education or unemployed but we speculate that non-payers will remain too few to significantly impact the financial stability of the project. The cross-subsidisation scheme should take account of a small proportion of children whose parents are likely to be unwilling to pay any amount. The amount generated from higher-priced spectacles can yield sufficient profits to compensate for the losses incurred from the subsidised and small proportion of free spectacles. The similar project in Timor demonstrated that one-third of the spectacles dispensed at higher prices accounted for two-thirds of total spectacle profits and made up for the losses incurred from subsidised and free spectacles. [14].

A suggested lower-priced spectacle at ₦2,000 (US$ 5.0) may be accessible to the disadvantaged population, such as those of lower household income and female and non-government employees. Our study indicated that the categories who are likely to pay for the higher-priced spectacles for their children are: (a) the male respondents, attributable to a higher socioeconomic status than females respondents (also reported by Anyabelechi et al. [23]) (b) government employees, which we speculate may be attributable to the regularity of their higher income and (c) those with higher education levels, which may be related directly to higher-income earners. We also hypothesised that higher-income earners might have an inclination toward glasses with greater aesthetic appeal. [18].

The many potential customers who can afford spectacles and the lack of competition also suggest a positive market potential for a cross-subsidisation scheme. Our previous study [17] at the local child eye clinics where the cross-subsidisation scheme was to be implemented also found that 9 in 10 children were first-time wearers. However, such a programme requires a long-term marketing and education scheme to gradually build sales growth seeing parents from the communities do not believe their child has a vision problem, [19] even though their child failed vision screening. In recent discussions with the Cross River State Eyecare Programme, the Department of Health identified a possible three-tier price structure within the cross-subsidisation scheme – low (₦2000 or US$5.0) for standard frames and lenses, mid (₦3600 or US$9.0) for upgraded frames and standard lenses, and high (₦4400 or US$11.0) for upgraded frames and lenses as reported by Yong et al. [17] and Chan et al. [18] Low-cost ready-made and clip-to-fit spectacles can be viable options for a lower-tier price without compromising the suggested quality standards for spectacles.

Precautions must be taken when deciding on these price structures based solely on our study findings. Firstly, the stated WTP may have been influenced by strategic, starting point, vehicle and information biases. Secondly, this was a selected sample of “active” caretakers whose actions indicated that they valued their services. We are uncertain whether all service users will value the service at the same level as the active caretakers. We recommend a more informed decision on pricing by piloting the pricing structure to determine if the stated demand at a given price can be translated into effective demand (willingness vs. ability to pay). Thirdly, our sample size was small and was drawn from a finite sample of those who attended the clinics during the 2 ½ month study period. Lastly, the sample was recruited from the eye clinics. Hence, this did not represent the demographic profiles of the population in Cross River State.

In these settings, frame design, material and quality are key factors influencing guardians when purchasing spectacles for their children, and female guardians or those with higher income prioritise frame quality. [18] To attract these parents with a higher income, we will need to focus on these features and offer them premium services. As much as they have low price elasticity, we need to ensure they do not feel penalised for being wealthier.

## Conclusions

The caretakers were willing to pay an average of ₦3600 (US$ 9.0) for a pair of children spectacles, which is slightly higher than the average price locally. Gender, government employment, education and earning a higher income were factors that influenced caretakers’ willingness to pay for their children’s spectacles. We will use the WTP study findings to plan a cross-subsidisation scheme that aim to improve the willingness to pay for children’s spectacles in CRS, Nigeria. Findings from this study will also enable the government and other interested organisations to develop an evidence-based, affordable spectacle scheme for children in other low- and middle-income countries.

## Data Availability

The datasets generated and/or analysed during the current study are available in the Queen’s University Belfast’s PURE repository, DOI: 10.17034/6f84899d-fafb-40dd-becd-998abea781f9.

## Abbreviations

WTP: Willingness to pay
CRS: Cross River State
